# Preliminary Findings From an Augmented Reality (AR) App Delivering Recovery‐Oriented Cognitive Therapy for Negative Symptoms in Schizophrenia

**DOI:** 10.1111/eip.70119

**Published:** 2026-01-06

**Authors:** Sunny X. Tang, Moein Foroughi, Aaron P. Brinen, Michael L. Birnbaum, Sarah A. Berretta, Leily M. Behbehani, John M. Kane, Edward Yoon, William F. Cronin

**Affiliations:** ^1^ North Shore Therapeutics, Inc. Rochester Minnesota USA; ^2^ Feinstein Institutes for Medical Research, Northwell Health Manhasset New York USA; ^3^ Zucker Hillside Hospital, Northwell Health Glen Oaks New York USA; ^4^ Donald and Barbara Zucker School of Medicine Hempstead New York USA; ^5^ Vanderbilt University Medical Center Nashville Tennessee USA; ^6^ Columbia University Vagelos College of Physicians and Surgeons New York New York USA; ^7^ New York State Psychiatric Institute New York New York USA; ^8^ School of Behavioral and Brain Sciences University of Texas at Dallas Richardson Texas USA; ^9^ Department of Psychology Yale University New Haven Connecticut USA

**Keywords:** app, augmented reality, CBT, CT‐R, digital health, negative symptoms, schizophrenia

## Abstract

**Background:**

NST‐SPARK is a novel digital therapeutic targeting negative symptoms in schizophrenia spectrum disorders (SSD). It is a smartphone application delivering recovery‐oriented cognitive therapy (CT‐R), via gamified augmented reality (AR) experiences. The primary objective was to determine the acceptability and feasibility of a single‐session prototype. Secondary objectives were to generate descriptive findings for changes in defeatist beliefs, self‐esteem and attitudes toward goal‐oriented activities.

**Methods:**

Twenty participants with schizophrenia or schizoaffective disorder enrolled. Participants completed self‐reports on the acceptability and feasibility of NST‐SPARK v.1.5 and provided open‐ended feedback. Self‐report scales on defeatist beliefs, self‐esteem and attitudes toward goal‐oriented activities were completed before and after participants were introduced to NST‐SPARK, and at 1‐week follow‐up.

**Results:**

Participants found NST‐SPARK to be feasible and acceptable, with an average response of ‘Agree’, indicating that the intervention met with the participants' approval and seemed implementable. Almost all participants (19 of 20) used the app on their own prior to the 1‐week follow up despite not being incentivised. We also observed changes in defeatist beliefs, self‐esteem and attitudes toward goal attainment consistent with intended improvements in these targeted areas. Participants made substantive progress toward identified goals in 90% of cases. The most common positive feedback was appreciation that the app got them going, and the most common negative feedback pointed out technical aspects that did not function properly.

**Conclusions:**

This preliminary, single‐arm, unblinded study of a single‐module prototype for NST‐SPARK found that the approach is generally acceptable and feasible for people with SSD and negative symptoms.

**Abbreviations**
AIM: acceptability of intervention measure
AR: augmented reality
ATT: attitudes toward behavioural change
BSES: Beck Self‐Esteem Scale
CT‐R: recovery‐oriented cognitive therapy
DBS: Defeatist Belief Scale
FDA: United States Food and Drug Administration
FIM: feasibility of intervention measure
NST: North Shore Therapeutics
SANS: Scale for the Assessment of Negative Symptoms
SCID‐5: Structured Clinical Interview for the Diagnostic and Statistical Manual of Mental Disorders, Fifth Edition
SSD: schizophrenia spectrum disorder
VR: virtual reality

## 1 Introduction

Effective treatment for negative symptoms in schizophrenia spectrum disorders (SSD) is a major unmet need. SSD are among the most devastating psychiatric disorders. Onset typically occurs in late adolescence or early adulthood and can severely disrupt completion of educational goals, obtaining and sustaining employment, and developing healthy relationships (Marshall et al. 2005; Perkins et al. 2005). While there are effective treatment options for positive psychotic symptoms, negative symptoms such as avolition, anhedonia, asociality and emotional flattening typically persist despite evidence‐based care (Carbon and Correll 2014; Azorin et al. 2014). In a meta‐analysis including over 12 000 individuals and 168 randomised‐controlled trials of interventions for negative symptoms in schizophrenia, few statistically significant effects on negative symptoms were evident, and none reached the threshold for clinically meaningful improvement (Fusar‐Poli et al. 2015). Prominent clinically significant negative symptoms are experienced by up to 60% of individuals with SSD and are often considered to be the greatest contributors to functional disability (Correll and Schooler 2020). Among the negative symptoms, avolition appears to be at the core, driving the greatest share of functional impairment and perhaps driving other negative symptoms (Strauss et al. 2021; Ahmed et al. 2022). Currently, there are no FDA‐approved medications or FDA‐cleared therapeutics specifically targeting avolition or any of the negative symptoms of schizophrenia.

NST‐SPARK is a novel digital therapeutic targeting negative symptoms. It is designed as a smartphone application that delivers recovery‐oriented cognitive therapy (CT‐R), via gamified augmented reality (AR) experiences, to provide experiential learning aimed at dismantling maladaptive beliefs.

CT‐R is an evidence‐based psychotherapy with efficacy in reducing avolition specifically, negative symptoms in general, and in improving functioning among individuals with SSD (Grant et al. 2017; Grant et al. 2012). In contrast to CBT for psychosis, CT‐R prioritises negative symptoms by focusing on patient engagement, correcting defeatist beliefs and reinforcing positive, recovery‐oriented beliefs and actions (Grant et al. 2018; Grant et al. 2014). It places greater emphasis on empowerment and engagement, and specifically targets avolition with high fidelity. Defeatist performance beliefs are overgeneralized negative thoughts about one's ability to accomplish tasks; they have been related to in‐the‐moment negative symptoms assessed via ecological momentary assessment (Luther et al. 2024) and contribute to the development and maintenance of negative symptoms. CT‐R attempts to correct defeatist beliefs by engaging individuals in rewarding activities while activating an adaptive mode of functioning. A randomised controlled trial (RCT) comparing CT‐R with standard treatment for 18 months in patients with SSD found significant reductions in avolition‐apathy negative symptoms (Cohen's *d* = −0.66, *p* = 0.01) and improvement in global functioning (Cohen's *d* = 0.56, *p* = 0.03) compared to treatment as usual (Grant et al. 2012). In a follow up RCT, between‐group differences for negative symptoms persisted 6 months after treatment was concluded (avolition‐apathy, Cohen's *d* = −0.66) (Grant et al. 2017).

However, while promising, CT‐R is limited by its reliance on highly trained clinicians. To maximise quality and access to care, clinicians need effective and customizable resources that can be used to elicit, identify and treat dysfunctional thought patterns and behaviours contributing to negative symptoms in SSD. In addition, in its original format, CT‐R is carried out with 50‐min weekly in‐person sessions over 18 months (Grant et al. 2017; Grant et al. 2012). This is not only resource‐intensive on the part of providers, but can also be burdensome for patients, not to mention necessitating a prolonged period before the clinical objectives can be achieved. Leveraging digital technology offers a potential solution to these limitations related to access, burden, and duration, as it allows individuals to complete multiple sessions within a more personalised timeframe—such as several sessions within a single week.

Immersive technologies have been deemed promising, safe and feasible for patients with SSD (Fernández‐Caballero et al. 2017; Kane et al. 2016), with no significant adverse effects demonstrated in a recent meta‐analysis on AR and VR interventions (Lundin et al. 2023). In one pilot study of a 3‐month VR intervention for negative symptoms of schizophrenia, 30 participants were randomised to receive treatment as usual or V‐NeST, which provided a combination of therapist‐supported psychoeducation, behavioural activation and insight development (Cella et al. 2022). V‐NeST was found to be feasible and acceptable, with no attributed adverse events, and a significant positive effect on goal attainment (*d* > 0.6); the effect on negative symptoms did not reach significance. For NST‐SPARK, AR was chosen for our implementation of CT‐R because it allows for delivery of an engaging experience while remaining anchored in the ‘real world’. This unique characteristic of AR was intended to encourage patients to interact with their surroundings instead of retreating to a simulated space, thereby potentially increasing the translation of lessons learned to the ‘real world’ where they are needed (Chicchi Giglioli et al. 2015). Unlike virtual reality (VR), which requires specialised hardware, AR can be implemented on smartphones, which increases its real‐world usability and scalability, and decreases implementation costs, an established barrier to the commercialization of several prior products. Meanwhile, it delivers a stimulating immersive experience that can interrupt negative feedback loops that perpetuate negative symptoms by transporting the patient to a novel environment. To date, there have been few studies completed using AR‐based therapies among patients with psychosis, but AR has been proposed as a platform for delivering treatment for auditory and visual hallucinations in schizophrenia (Fernández‐Caballero et al. 2017), and has been shown to be promising in other applications such as rehabilitative training (Tan et al. 2022), autism spectrum disorder (Liu et al. 2017; Antão et al. 2020), and specific phobias (Botella et al. 2016).

The simulated experiences in AR can be used to engage reward circuitry and motivate users to change behaviour by incorporating elements of gaming (Gay et al. 2016). Gamification involves using game‐based elements and game thinking to motivate action and promote learning and engagement. The global phenomenon Pokémon Go, for example, demonstrated an increase in physical activity and a decrease in psychological distress in non‐psychiatric populations (Althoff et al. 2016; Howe et al. 2016). The popular language learning app, Duolingo, uses gamification elements in language learning and has shown to positively affect learners' behaviour, commitment, and motivation (Shortt et al. 2023). Game‐like digital environments hold promise to improve negative symptoms through activating reward circuitry and eliciting positive experiences and emotions in people with SSD by leveraging proven design elements like points, rewards and progress tracking (Välimäki et al. 2021; Litvin et al. 2020), thereby creating a neuropsychological state that may be conducive to treatment.

In this study, we assessed a prototype of NST‐SPARK (NST‐SPARK v.1.5) in 20 volunteers with SSD and clinically significant negative symptoms. NST‐SPARK v.1.5 delivers a single session of CT‐R, along with two AR experiences. Participants repeated the session over a 1‐week period. The primary objective was to determine the acceptability and feasibility of this approach. Secondary objectives were to generate descriptive findings for changes in defeatist beliefs, self‐esteem, and attitudes toward goal‐oriented activities.

## 2 Methods

### 2.1 Participants

Recruitment was completed from volunteers from prior market research studies as well as online through referrals and social media advertisements (Reddit, Twitter and Facebook). Interested potential participants completed an initial screening visit to determine eligibility. Diagnosis of schizophrenia or schizoaffective disorder was confirmed by the Structured Clinical Interview for DSM‐5 (SCID‐5) (First 2015). Additional inclusion criteria included age 18–65 years, all genders, races, and ethnicities, dependable access to stable Wi‐Fi and a device with videoconferencing ability, able to provide informed consent, proficient in English, and clinically significant negative symptoms (rating of 2 or higher on the Avolition/Apathy or Anhedonia/Asociality) subscale of the Scale for the Assessment of Negative Symptoms (SANS) (Andreasen 1984). Exclusion criteria included acute safety concerns, recent change in level of care (medication changes within 4 weeks or acute psychiatric care within 12 weeks), substance‐induced psychotic disorder, or significant cognitive or physical limitations that prevented the participant from being able to operate NST‐SPARK. Eligible participants provided informed consent via a secure digital platform, Zoho. All study procedures were approved by the BRANY IRB #23–02–339‐1483. This study is registered on ClinicalTrials.gov #NCT06653829.

Twenty participants with schizophrenia or schizoaffective disorder were enrolled, with a range of demographic and socioeconomic status and treatment settings (Table 1). Compensation was provided at study conclusion; participants had the choice between keeping the study iPhone (a refurbished second‐generation iPhone SE) or sending it back for a $100 gift card of their choice.

**TABLE 1:** Participant characteristics.

Demographic characteristics	
*N*	20
Age: years (SD)	31.6 (8.1)
Sex: *n* (%)	
Male	8 (40%)
Female	12 (60%)
Gender: *n* (%)	
Man	8 (40%)
Woman	8 (40%)
Non‐binary	4 (20%)
Race: *n* (%)	
White/Caucasian	9 (45%)
Black/African American	5 (25%)
Asian	4 (20%)
Other	1 (5%)
Multiple	1 (5%)
Ethnicity: *n* (%)	
Not Hispanic	18 (90%)
Hispanic	2 (10%)
Education: years (SD)	14.9 (2.4)
Socioeconomic status	
Maternal education: years (SD)	14.6 (2.7)
Paternal education: years (SD)	17.4 (19.8)
Employment status: *n* (%)	
Full‐time employed	4 (20%)
Part‐time employed	3 (15%)
Unemployed	13 (65%)
Marital status: *n* (%)	
Never married	17 (85%)
Married	2 (10%)
Divorced	1 (5%)
Clinical status	
Diagnosis: *n* (%)	
Schizophrenia	9 (45%)
Schizoaffective disorder	11 (55%)
Insurance status: *n* (%)	
Uninsured	1 (5%)
Medicare	3 (15%)
Medicaid	6 (30%)
Private	9 (45%)
Other	1 (5%)
Treatment setting: *n* (%)	
Private clinic	11 (55%)
Academic program	1 (5%)
Psychosis specialty care	4 (20%)
Community MH center	3 (15%)
Other	1 (5%)

Abbreviations: *n*, count; SD, standard deviation.

### 2.2 Study Procedures

The study consisted of 3 remote visits: an initial screening visit, followed by Visit 1, and a 1‐week follow‐up at Visit 2. All research procedures were done remotely, through video conferencing, by two centralised trained assessors. The screening visit consisted of establishing that the potential participant met inclusion/exclusion criteria and obtaining informed consent. The study iPhone was then provided to participants prior to Visit 1. Upon receiving the iPhone, participants completed Visit 1 remotely with the same assessor, where they were introduced to the application, using it under observation by the assessor, and completed questionnaires before and after the experience. Participants were given the recommendation of repeating NST‐SPARK in the intervening week before Visit 2, but there was no incentive given for doing so. Participants were told that the session could be repeated on the app, but no minimum usage was required, and no additional remuneration was provided for additional app engagement. A mid‐week check‐in was conducted by the same research assistant to address any technical issues and to support continued access and use of the app, but no instruction was given to use the app. Visit 2 occurred 1 week after participants were introduced to NST‐SPARK, where participants completed additional questionnaires and reported on their experiences with the app during the week.

### 2.3 NST‐SPARK v.1.5

**FIGURE 1:**
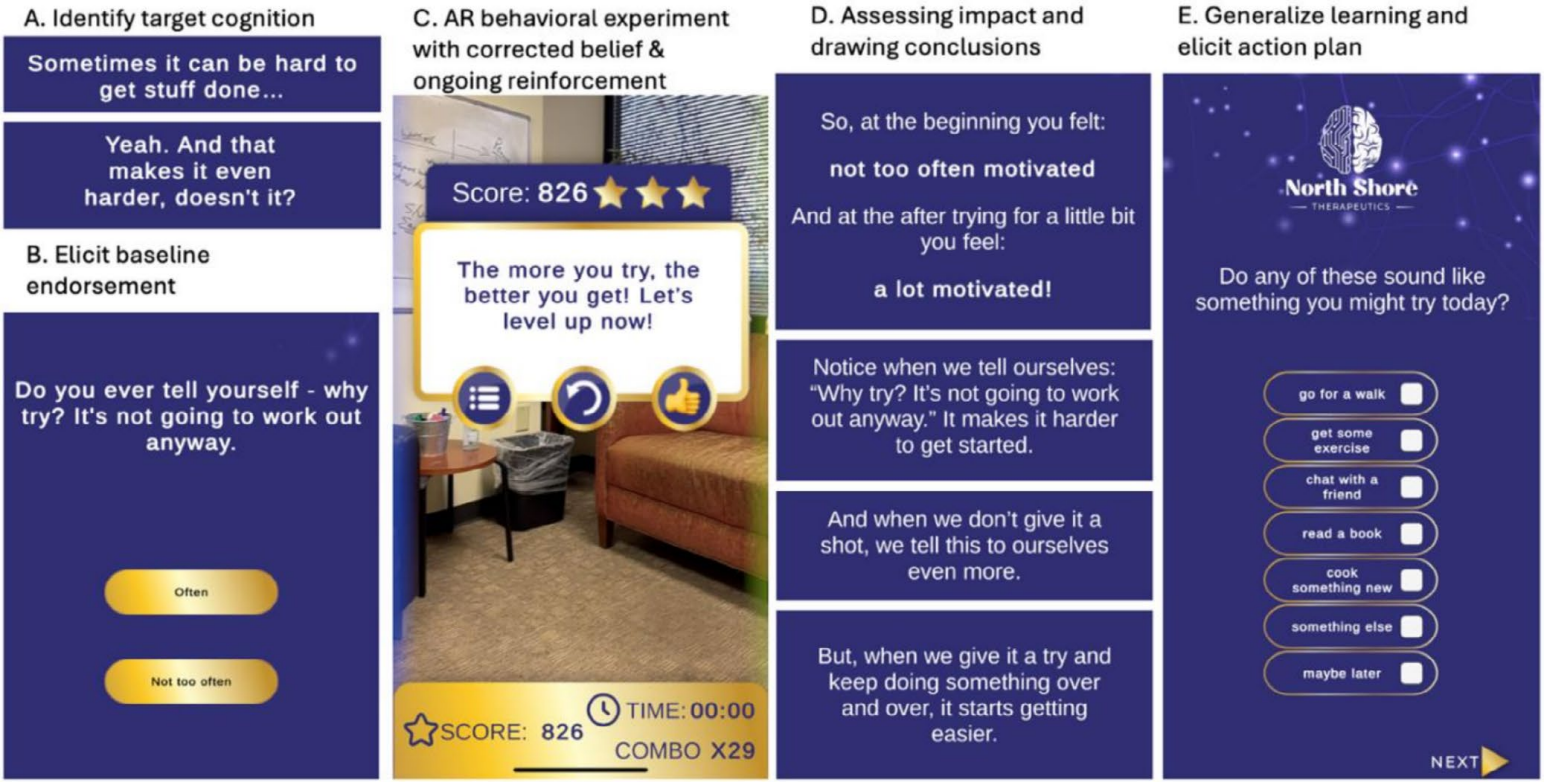
NST‐SPARK v.1.5 user experience.

NST‐SPARK v.1.5 was designed by psychiatrists with extensive expertise in psychosis intervention (SXT, MLB and JMK) and a psychologist (APB) who was a co‐inventor of CT‐R. This version also incorporates input obtained via focus groups from people with lived experience with psychosis who were exposed to an earlier minimally‐viable prototype. Participants received an unlocked second‐generation iPhone SE with the NST‐SPARK v.1.5 application. NST‐SPARK v1.5 delivers a single therapeutic session based on CT‐R (Figure 1). The app leads participants to (A/B) elicit targeted defeatist beliefs, (C) engage in a brief (1–2 min) gamified AR experience where participants received encouraging prompts as they attempted to sort flying objects based on their colour, (D) draw conclusions based on the experience and (E) generalise the lesson to positive real‐life pursuits. A second AR experience was also included, interspersed with CT‐R based prompts. In the second AR experience, cartoon visual stimuli (bottles) were placed around the AR space. Participants would move around their physical space to collect the bottles. In total, one session via NST‐SPARK v.1.5 took 10–15 min to complete and did not require therapist involvement.

### 2.4 Assessments

At Visit 1, participants completed self‐report questionnaires both before and after completing NST‐SPARK. Questionnaires included: the Defeatist Belief Scale (DBS) (Grant and Beck 2009), a 5‐item measure of maladaptive beliefs scored on a 7‐point Likert scale (1 = Totally Agree to 7 = Disagree Totally) the Beck Self‐Esteem Scale (BSES) (Thomas et al. 2018), a 18‐item measure assessing self‐esteem on a 10‐point scale ranging from one descriptor to its opposite (e.g., 1 = Successful to 10 = Unsuccessful; or 1 = Smart to 10 = Dumb); and a custom scale for attitudes toward behavioural change (NST‐ATT; Appendix A). Briefly, NST‐ATT asked participants to identify two tasks or goals they wanted to accomplish, one that is relatively easy and achievable (Task A) and one that is very important to them (Task B). Participants then rated their level of intention to accomplish the task, and their confidence in being able to do so each on 5‐point Likert scales (1 = Definitely Disagree to 5 = Definitely Agree). Upon completion of the NST‐SPARK session, participants repeated the DBS, BSES and NST‐ATT. In addition, participants responded to the acceptability of intervention measure (AIM) and feasibility of intervention measure (FIM) self‐report scales (Weiner et al. 2017), both of which use a 5‐point Likert scale (1 = Completely Disagree to 5 = Completely Agree). Finally, participants completed a semi‐structured interview eliciting qualitative feedback on their experience with NST‐SPARK v.1.5 (Appendix B).

Visit 2 began with a semi‐structured interview regarding their experiences with the app over the last week (Appendix B). Participants were then asked to repeat the DBS and the BSES, as well as report on any progress made toward the tasks identified during Visit 1 on the NST‐ATT. We tried to minimise any potential effect of social pressure by having participants complete the self‐report assessments (AIM, FIM, DBS and BSES) directly through an anonymous web interface, rather than verbally providing answers to the assessors.

### 2.5 Statistical Analysis

As this is a preliminary study with the primary objective of determining the acceptability and feasibility of NST‐SPARK v.1.5. The results are primarily represented with straightforward descriptive statistics (mean, standard deviation [SD] and percentages), as well as quotes and qualitative descriptions from open‐ended participant feedback. We also report Cohen's *d* effect sizes for changes in the exploratory outcomes on defeatist beliefs, self‐esteem, and attitudes toward goal‐oriented activities. All analyses were done in R v.4.2.0 (R Core Team 2020).

## 3 Results

### 3.1 Acceptability and Feasibility of NST‐SPARK v.1.5

In general, participants found NST‐SPARK v.1.5 to be feasible and acceptable, responding with an average response of ‘Agree’ to the AIM and FIM questionnaires, indicating that the intervention was found to meet with the participants' approval and seemed implementable. Table 2 summarises the participants' responses to the AIM and FIM questionnaires. Almost all participants (19 of 20) self‐reported using the app on their own prior to the 1‐week follow up (frequency and duration was not recorded).

**TABLE 2:** Self‐report responses for acceptability and feasibility of NST‐SPARK.

	Visit 1—Timepoint 2	Effect size	Visit 2—Timepoint 3
	After NST‐SPARK	T2—T3	1‐week follow up
NST‐SPARK meets my approval	3.9 (0.6)		3.9 (0.9)
NST‐SPARK is appealing to me	3.6 (1.0)		3.9 (0.8)
I like NST‐SPARK	3.8 (0.7)		3.9 (0.9)
I welcome NST‐SPARK	3.9 (0.7)		4.2 (0.7)
Mean AIM score	3.8 (0.6)	0.26	4.0 (0.7)
NST‐SPARK seems implementable	3.8 (0.9)		3.9 (0.8)
NST‐SPARK seems possible	4.1 (0.6)		4.2 (0.9)
NST‐SPARK seems doable	3.8 (0.9)		4.0 (0.9)
NST‐SPARK seems easy to use	3.5 (1.0)		3.9 (0.9)
Mean FIM score	3.8 (0.7)	0.30	4.0 (0.6)

Abbreviations: AIM, acceptability of intervention measure; FIM, feasibility of intervention measure.

In addition, the participants responded to open‐ended feedback questions in a generally positive way. Overall, the approach was found to be acceptable, with the main limitations identified in the mechanisms of the NST‐SPARK v.1.5 prototype. Their first reactions to the app ranged from largely positive related to the gamification (‘sparkly theme’ ‘It is pretty cool, I like that it was interactive.’ ‘I really liked actual activity’) and technology (‘I liked the AR’) to some negative reactions related to program bugs (‘the tapping did not work’) and not knowing the purpose of the app. When asked what they liked most, many responded to the interactive nature (‘It says things to validate what you are thinking’, ‘I like that it talks to me’, “interactive nature, moving around’, ‘to find the bottles around the room was fun’, ‘it was very interactive, like playing a game’), the nov elty, (‘it was different a little bit, I have never done it before’, ‘It was fun’), the therapeutic message (‘I liked the idea of creating energy through action. The basic idea of it was fun’), and the sense it helped (‘Could help with focus. Liked the sustained keeping going, not tiring out.’; ‘I like that it works’, ‘I liked the actual activity, it was really engaging and fun. Intriguing. I am curious how it will help with motivation. It helped me get out of bed so that was nice’). Most responses to what was liked least revolved around user interfaces with 4 participants mentioning issues with responding to tapping and other design limitations (‘the second level was too long’, ‘it could stop going so fast towards the end, for what you are supposed to read’, ‘needs more sound effects’, ‘I did not like how it was talking to me’). Participants also brought up the limited nature of the prototype (‘it is repetitive,’ ‘could engage in more than one way’); One participant found it too difficult, and one participant brought up concerns related to accessibility for people with mobility issues. When asked if they would use it if prescribed by a professional, largely participants indicated they would use it (18 of 20): some with enthusiasm (‘Yes. I think it could reach a point where you did want to play it three or more times a week’), some due to power of the prescriber (‘Yes, because it is not that challenging. I trust my doctor.’), and a small proportion of participants indicated they would not be willing to use the app because they did not find it useful (2 of 20 participants).

Negative effects reported in the feedback include 2 people who reported feeling mild dizziness but were able to complete the session as well as completing additional sessions during the week. They felt that either sitting down for the activities or using a larger screen would ameliorate these effects. The single participant who did not use the app during the intervening week reported that they felt ‘spooked’ by the augmented reality environment and had pre‐existing delusions regarding being monitored. One additional participant reported that the app also triggered her technology‐related paranoid delusions, but nevertheless used it daily and found it ‘helpful and nice to do in the morning to jumpstart the day.’ The other 16 participants did not report any adverse effects.

### 3.2 NST‐SPARK Effects on Defeatist Beliefs, Self‐Esteem and Goal Attainment

We observed shifts in attitudes toward goal attainment, defeatist beliefs and self‐esteem in the intended directions. Overall change in defeatist beliefs was small (Table 3), with total score on the DBS remaining stable before and after using NST‐SPARK during Visit 1, and then improving slightly at the 1‐week follow‐up (Cohen's *d* = 0.12 relative to initial assessment). The largest shifts occurred in ‘If I do not do as well all the time, people will not respect me’ (Cohen's *d* = 0.23) and ‘If I fail partly, it is as bad as being a complete failure’ (Cohen's *d* = 0.21).

**TABLE 3:** Changes in defeatist beliefs.

	Visit 1—Timepoint 1	Effect Size	Visit 1—Timepoint 2	Effect Size	Visit 2—Timepoint 3	Effect Size
	Before NST‐SPARK	T1—T2	After NST‐SPARK	T2—T3	1‐week follow up	T1—T3
If I do not do as well all the time, people will not respect me	3.4 (1.8)		3.5 (1.7)		3.8 (1.6)	
I cannot be happy unless most people I know admire me	4.6 (1.6)		4.3 (1.9)		4.4 (1.5)	
If I do not do as well as other people, it means I am an inferior human being	4.5 (1.8)		4.7 (1.8)		4.4 (1.7)	
If you cannot do something well, there is little point in doing it at all	4.8 (1.9)		4.8 (1.9)		5.1 (1.4)	
If I fail partly, it is as bad as being a complete failure	4.7 (1.8)		4.5 (1.6)		5.0 (1.6)	
DBS total score	21.9 (6.6)	0.00	21.9 (8.2)	0.11	22.7 (6.4)	0.12

*Note:* Participants indicated their agreement or disagreement with the statements shown. Higher scores represent *disagreement* with the statements, that is, *lower* defeatist beliefs. For each timepoint, the mean score with deviation is shown. Effect sizes are calculated with the formula for Cohen's d.

Abbreviation: DBS, Defeatist Beliefs Scale.

There were larger effect sizes for self‐esteem (Table 4), with improvements in the total score for BSES before and after using NST‐SPARK (Cohen's *d* = −0.09) and then again at the 1‐week follow‐up (Cohen's *d* = −0.12), for an overall shift of effect size −0.21. The largest overall shifts occurred in the endorsement of being ‘Desirable/Undesirable’ (Cohen's *d* = −0.55) and being ‘Successful/Unsuccessful’ (Cohen's d = −0.37).

**TABLE 4:** Changes in Self‐Esteem.

	Visit 1—Timepoint 1	Effect Size	Visit 1—Timepoint 2	Effect Size	Visit 2—Timepoint 3	Effect Size
	Before NST‐SPARK	T1—T2	After NST‐SPARK	T2—T3	1‐week Follow Up	T1—T3
Successful/Unsuccessful	5.9 (2.2)		5.0 (2.2)		5.0 (2.6)	
Attractive/Unattractive	5.8 (2.4)		6.1 (2.5)		5.5 (2.6)	
Popular/Unpopular	7.1 (2.6)		6.8 (2.6)		6.8 (3.1)	
Independent/Dependent	5.1 (2.7)		4.9 (2.3)		4.8 (2.4)	
Honest/Dishonest	3.8 (2.8)		3.5 (2.5)		3.0 (2.1)	
Desirable/Undesirable	6.5 (2.2)		5.7 (2.1)		5.3 (2.1)	
Strong/Weak	5.0 (2.4)		5.2 (2.4)		4.5 (2.2)	
Smart/Dumb	4.1 (2.0)		4.1 (2.6)		4.1 (2.4)	
Powerful/Powerless	5.4 (2.1)		5.4 (2.2)		5.5 (2.5)	
Lovable/Unlovable	5.0 (2.4)		5.1 (2.3)		4.7 (2.1)	
Pleasant/Unpleasant	4.2 (2.4)		4.0 (2.4)		3.6 (2.3)	
Efficient/Inefficient	4.7 (2.3)		5.0 (2.8)		4.9 (2.8)	
Responsible/Irresponsible	4.5 (2.4)		4.2 (2.4)		4.1 (2.5)	
Generous/Selfish	3.3 (1.8)		3.5 (2.1)		3.4 (2.0)	
Worthwhile/Worthless	4.5 (2.0)		4.3 (2.3)		4.6 (2.5)	
Interesting/Boring	4.8 (2.4)		4.5 (2.4)		4.2 (2.4)	
Knowledgeable/Ignorant	3.9 (2.1)		4.0 (2.0)		3.6 (2.0)	
Good/Bad	4.2 (2.5)		4.0 (2.5)		3.9 (2.5)	
BSES total score	87.8 (28.0)	−0.09	85.2 (30.2)	−0.12	81.5 (33.8)	−0.21

*Note:* Participants indicated their agreement along a Likert scale for each pair of terms as applied to themselves. Higher scores represent more agreement with the *negative* term, that is, *lower* self‐esteem. For each timepoint, the mean score with deviation is shown. Effect sizes are calculated with the formula for Cohen's d.

Abbreviation: BSES, Beck Self Esteem Scale.

With regard to attitudes toward goal‐oriented tasks, participants reported increased intention to complete the tasks (Cohen's *d* = 0.13) as well as increased confidence in their ability to do so (Cohen's *d* = 0.35) after using a single session of NST‐SPARK during Visit 1 (Table 5). For Task A, the relatively easy and achievable task, participants identified tasks related to cleaning or organisation (*n* = 4), tasks related to applying for jobs or positions (*n* = 4), laundry (*n* = 3), cooking (*n* = 2), grocery shopping (*n* = 1), art projects (*n* = 2), reading (*n* = 1), exercise (*n* = 1), and showering regularly (*n* = 1). For Task B, the task that was most important to them, participants identified tasks related to getting a job or career (*n* = 6), health and exercise (*n* = 5), cleaning (*n* = 3), artistic projects (*n* = 3), and educational goals (*n* = 2). At 1‐week follow‐up, the overwhelming majority of participants (90%) reported making progress in at least one of the tasks they had identified.

**TABLE 5:** Attitudes toward goal‐oriented activities.

A. Intention and confidence in accomplishing tasks		
	Visit 1—Timepoint 1	Visit 1—Timepoint 2
	Before NST‐SPARK	After NST‐SPARK
I intend to work toward accomplishing this task in the next week. (Task A)	4.5 (0.9)	4.4 (1.3)
I intend to work toward accomplishing this task in the next week. (Task B)	3.8 (1.4)	4.2 (1.0)
I intend to work toward accomplishing this task in the next week. (Mean of Task A + Task B)	4.2 (0.9)	4.3 (0.9)
I am confident that I am able to accomplish this task. (Task A)	3.8 (1.2)	4.1 (1.2)
I am confident that I am able to accomplish this task. (Task B)	3.4 (1.3)	3.9 (1.0)
I am confident that I am able to accomplish this task. (Mean of Task A + Task B)	3.6 (1.2)	4.0 (1.0)

B. Progress toward accomplishing tasks	
Progress at timepoint 3	
Task A: *n* (%)	
Did not make progress	5 (25%)
Made progress	15 (75%)
Task B: *n* (%)	
Did not make progress	7 (35%)
Made progress	13 (65%)
Overall: *n* (%)	
Did not make progress on either task	2 (10%)
Made progress on one task	8 (40%)
Made progress on both tasks	10 (50%)

## 4 Discussion

### 4.1 Key Findings

NST‐SPARK v.1.5 is a single‐module prototype for an AR app that employs gamification and the therapeutic principles of recovery‐oriented cognitive therapy to target negative symptoms in schizophrenia. The primary objective of this study was to establish its acceptability and feasibility, and to inform further development of the app, if warranted. Secondary objectives were to explore changes in the targets of the intervention: defeatist beliefs, self‐esteem (i.e., beliefs about the self) and attitudes and progress toward goal‐oriented tasks.

In general, the primary goals were achieved. Participants responded mostly favourably to the app, and particularly to the AR and gamification approach, as well as the therapeutic goal of reducing defeatist beliefs and avolition. They also used the app during the intervening 1‐week period despite not being provided with additional incentives to do so. Some main limitations identified had to do with the operation of the app itself and features not working as intended. We believe that these limitations can be readily addressed with further investment in a more complete version of NST‐SPARK. There were also a few cases of negative effects with mild dizziness that did not impede use in two participants, and two participants with preexisting paranoid ideations surrounding technology (one participant was able to use the app and found it helpful; one participant was not able to use the device). We plan to address the dizziness through refining the design of the app and making it available on tablet devices in the future. We will also add prominent disclosures that the app does not record any information on the participants' surroundings.

Additionally, while we cannot draw causal relationships from NST‐SPARK due to the lack of a control group for this preliminary study, we did observe changes in defeatist beliefs, self‐esteem, and goal‐oriented tasks that were in the intended and hoped‐for directions. This is consistent with, though does not directly support, the premise that NST‐SPARK targets defeatist beliefs related to negative symptoms. The possibility of defeatist beliefs improving after a brief intervention is also consistent with earlier work where 35 participants with schizophrenia were randomised to perform a card sorting task either with or without a guided success experience (an analogue of the AR experience in NST‐SPARK) (Grant et al. 2018); participants who received a guided success experience endorsed significant lower defeatist beliefs on the DBS and higher self‐esteem on the BSES. The goal of NST‐SPARK is to elicit this effect reliably and repeatedly, integrating the guided success experiences during the AR activities with cognitive restructuring and goal setting. Remarkably, in this study, 90% of participants made concrete progress toward at least one goal that they had identified during the intervention.

To our knowledge, there are no other apps using immersive technology to deliver a treatment for negative symptoms of schizophrenia. While there are other clinical trials underway, we are not aware of any published studies to which NST‐SPARK can be compared.

### 4.2 Limitations & Future Considerations

The single‐arm, unblinded design of this study is associated with obvious limitations but was a key step in the design process to provide impetus for further development of NST‐SPARK. Feedback obtained from the participants will be incorporated along with active engagement from individuals with lived experience with psychosis to produce a NST‐SPARK v.2.0 that will deliver the full treatment for negative symptoms. We are planning to deliver a 12‐week course with weekly modules focusing on different defeatist beliefs, and 3 unique but coordinated sessions per module. This next iteration will be tested in a randomised controlled trial over a more extended period, and the effects on negative symptoms will be quantified and reported. Negative symptoms were not included as outcome measures because meaningful changes in these symptoms typically require a more intensive or sustained intervention. As negative symptoms are considered persistent by definition and less responsive to brief interventions, it is unlikely that a short‐term study would detect measurable improvement. Because of the earlier work where participants demonstrated lower defeatist beliefs and higher self‐esteem after being randomised to a guided success experience on a card‐sorting task, we *did* hypothesize a possible shift in defeatist performance beliefs even with a single session of NST‐SPARK. We attempted to capture this with the same scales used in the cited study (DBS & BSES), though we acknowledge that these scales have not been psychometrically validated for repeated use in this way. In addition, NST‐ATT was created by the authors here to assess what we felt were relevant secondary outcomes (attitudes and steps taken toward goal‐oriented activities); there were no equivalent measures available to our knowledge, and as such, this scale is not validated beyond the findings reported here.

In the design of NST‐SPARK v.2.0, we will take steps to ameliorate the adverse effects reported from dizziness and pre‐existing paranoid ideations, but we also acknowledge that the app may not be ultimately suitable for all individuals. We also plan to electronically capture the frequency, timing and duration of app use in future studies. To enable access for the large number of people with schizophrenia who do not have access to regular psychotherapy, NST‐SPARK is intended as a stand‐alone prescription digital therapeutic and will continue to be administered independently without therapist intervention. However, we do plan to build a clinician‐portal where patients can elect to share their progress with their regular treatment team.

The participants in this study were relatively diverse with regard to race, ethnicity, gender and socioeconomic background, including some participants on Medicaid/Medicare and those in community mental health programs. However, because this study was conducted remotely, the participants in this study may be enriched for individuals who had greater access to and familiarity with technology than average and may be more motivated than average. Future studies will also include recruitment directly from treatment centers and efforts will be made to accommodate participants who may have lower access to technology.

## 5 Conclusions

NST‐SPARK is a mobile phone application that uses gamified AR experiences to delivery recovery‐oriented cognitive therapy (CT‐R). This preliminary, single‐arm, unblinded study of a single‐module prototype found that this approach is generally acceptable and feasible for people with SSD who have negative symptoms. Engagement of the intended target of defeatist beliefs and the ultimate effectiveness of NST‐SPARK for negative symptoms require confirmation in future randomised controlled trials. Overall, we find that NST‐SPARK is based on a promising approach and further development is warranted.

## Funding

This study was funded by North Shore Therapeutics.

## Conflicts of Interest

S.X.T., A.P.B., M.L.B., S.A.B., L.M.B., and J.M.K. are affiliated as advisors and consultants for North Shore Therapeutics and are also employed full‐time by academic institutions as described above. North Shore Therapeutics does not have any formal relationship with any of the above academic institutions. E.Y. and W.C. are full‐time employees of North Shore Therapeutics. In addition, S.X.T. received research funding and serves as a consultant for Winterlight Labs, is on the advisory board and owns equity for Psyrin, and serves as a consultant for Catholic Charities Neighborhood Services and LB Pharmaceuticals. J.M.K. has received consulting fees or Honoria for lectures from Alkermes, Boerhinger Ingelheim, Cerevel, Click Therapeutics, Intracellular Therapies, H. Lundbeck, HLS, Janssen, Johnson and Johnson, Merck, Minerva, Neurocrine, Newron, Otsuka, Roche, Saladax and Teva. He is a shareholder in Cerevel, HealthRyhthms, LB Pharma, North Shore Therapeutics and The Vanguard Research Group. He has received grant support from H. Lundbeck, Otsuka, Merck, Sunovion and Valera.

## Data Availability

The data that support the findings of this study are openly available in Github at https://github.com/NST‐Spark/NST_Spark1b.

## Appendix A Attitudes Toward Behavioural Change (NST‐ATT)

(Likert scale applied to agree/disagree questions below.)

Part 1 (before NST‐SPARK).

1. Is there anything on your to‐do list currently, any projects you would like to work on, or anything you would like to accomplish in the near future?
  - (Probe as needed. If not much content) Is there anything that your family or friends have been encouraging you to do, but that you haven't done?
  - (If no response or unsure–ask about chores, meeting up with a friend or family member, improving health, getting a job or going to school.)
2. Which of these tasks seems the easiest to tackle? TASK A.
3. Which of these is the most important to you? TASK B (get a different response if they initially answer the same task).
4. Thinking about TASK A, how much would you agree or disagree with the following?
  - I intend to work toward accomplishing this task in the next week.
  - I am confident that I am able to accomplish this task.
5. Thinking about TASK B? how much would you agree or disagree with the following?
  - I intend to work toward accomplishing this task in the next week.
  - I am confident that I am able to accomplish this task.

Part 2 (after NST‐SPARK).

1. Before, you said that TASK A was something you would like to accomplish. How much would you agree or disagree with the following, now?
  - I intend to work toward accomplishing this task in the next week.
  - I am confident that I am able to accomplish this task.
2. What would be the first step to accomplishing TASK A? (Guide the participant to a concrete small step, for example, put all the dirty clothes in the hamper; sign up for a handshake account; make a grocery list).
3. Before, you said that TASK B was something you would like to accomplish. How much would you agree or disagree with the following, now?
  - I intend to work toward accomplishing this task in the next week.
  - I am confident that I am able to accomplish this task.
4. What would be the first step to accomplishing TASK B?

Part 3 (1wk follow‐up).

1. Last time, you said that TASK A was something you would like to accomplish. Did you make any progress on it over the past week? YES/NO (description).
2. Last time, you said that TASK B was something you would like to accomplish. Did you make any progress on it over the past week? YES/NO (description).

## Appendix B NST‐SPARK Semi‐Structured Feedback Interview.

Assessor/Moderator to elaborate or ask follow up questions as needed.

1. Visit 1.
  - What are your first impressions?
  - What do you like most about the app?
  - What do you like least about the app or what are some areas that need to be improved?
  - Did you experience any negative effects from using the app? For example, dizziness, vertigo or changes in your symptoms?
  - Do you think you did be able to use this app from a technical perspective? Does it seem simple enough to operate? If no, which parts seemed confusing or difficult?
  - (Imagine that we added similar but more elaborate experiences for a 12‐week long experience, and also provided guides for reaching your goals in the real world.) Would you use this app if your doctor prescribed it for you? What makes you say that?
  - If there were other activities like the one you saw, what would make it fun and engaging for you?
  - Is there any other feedback that you did like to provide to help us make this app better?
2. Visit 2.
  - Did you get a chance to use the app during the week? If yes, how was your experience? If no, what got in the way?
  - Did you tell anyone about the app? Show it to friends or family?
  - Did you experience any negative effects from using the app? For example, dizziness, vertigo or changes in your symptoms?
  - (Imagine that we added similar but more elaborate experiences for a 12‐week long experience, and also provided guides for reaching your goals in the real world.) Would you use this app if your doctor prescribed it for you? What makes you say that? If yes, how often do you think you would use it on a weekly basis?
  - Do you have any more thoughts about what would make it fun and engaging for you?
  - Is there any other feedback that you did like to provide to help us make this app better?
